# Supplementary material for: Venomix: a simple bioinformatic pipeline for identifying and characterizing toxin gene candidates from transcriptomic data
Source: PeerJ. 2018 Jul 31;6:e5361. doi: 10.7717/peerj.5361 (PMC6074769; doi:10.7717/peerj.5361)
Supplement: Supplemental Information 3 [file peerj-06-5361-s003.gz › FinalOutput_E-20/Venom_prothrombin_activator_pseutarin-C_catalytic_subunit_12/finaltree.pdf]

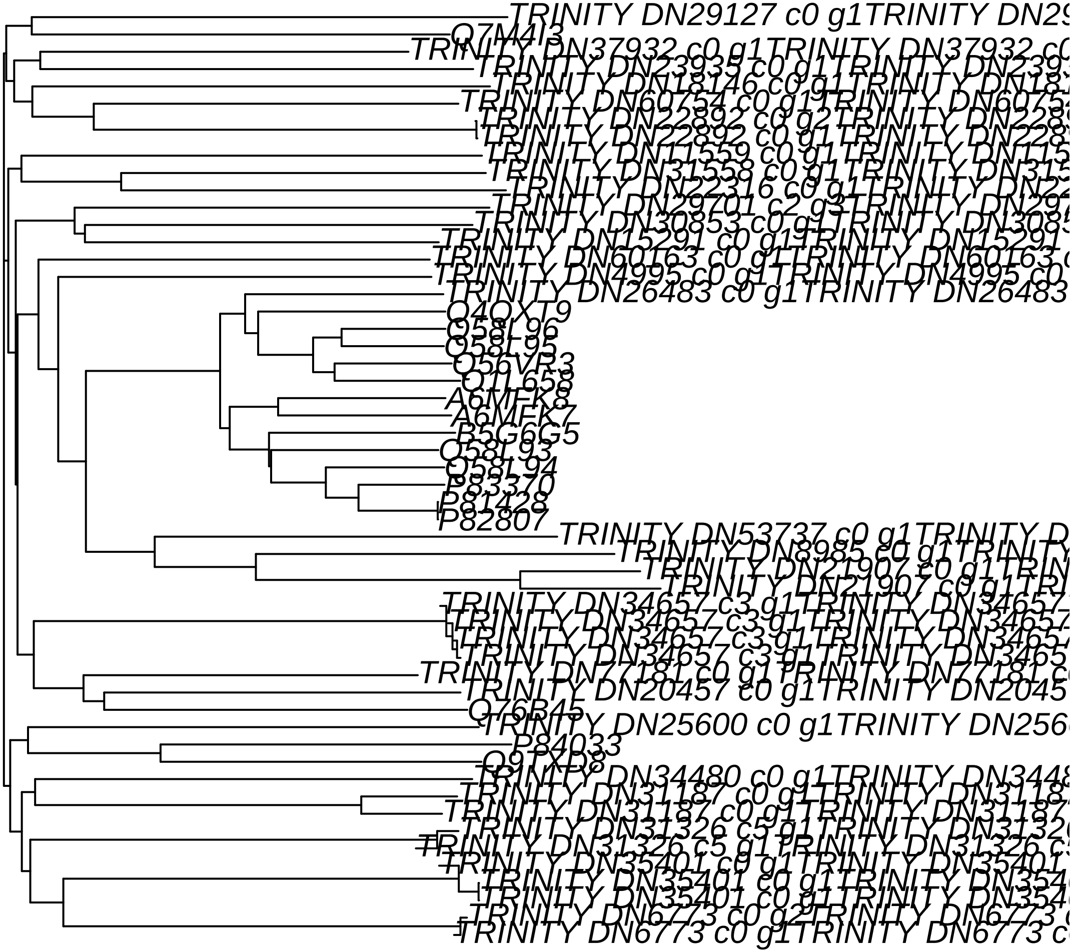
TRINITY DN29127 c0 g1TRINITY DN29
Q7M4I3
TRINITY DN37932 c0 g1TRINITY DN37932 c0
TRINITY DN23935 c0 g1TRINITY DN2393
TRINITY DN18146 c0 g1TRINITY DN18
TRINITY DN60754 c0 g1TRINITY DN6075
TRINITY DN22892 c0 g2TRINITY DN228
TRINITY DN22892 c0 g1TRINITY DN228
TRINITY DN11559 c0 g1TRINITY DN115
TRINITY DN31558 c0 g1TRINITY DN315
TRINITY DN22316 c0 g1TRINITY DN22
TRINITY DN29701 c2 g3TRINITY DN297
TRINITY DN30853 c0 g1TRINITY DN308
TRINITY DN15291 c0 g1TRINITY DN15291
TRINITY DN60163 c0 g1TRINITY DN60163
TRINITY DN4995 c0 g1TRINITY DN4995 c0
TRINITY DN26483 c0 g1TRINITY DN26483
Q4QXT9
Q58L96
Q58L95
Q56VR3
Q1L658
A6MFK8
A6MFK7
B5G6G5
Q58L93
Q58L94
P83370
P81428
P82807
TRINITY DN53737 c0 g1TRINITY D
TRINITY DN8985 c0 g1TRINITY
TRINITY DN21907 c0 g1TRIN
TRINITY DN21907 c0 g1TRI
TRINITY DN34657 c3 g1TRINITY DN34657
TRINITY DN34657 c3 g1TRINITY DN3465
TRINITY DN34657 c3 g1TRINITY DN3465
TRINITY DN34657 c3 g1TRINITY DN3465
TRINITY DN7181 c0 g1TRINITY DN7181 c
TRINITY DN20457 c0 g1TRINITY DN2045
Q76B45
TRINITY DN25600 c0 g1TRINITY DN256
P84033
Q9TXP8
TRINITY DN34480 c0 g1TRINITY DN344
TRINITY DN31187 c0 g1TRINITY DN3118
TRINITY DN31187 c0 g1TRINITY DN3118
TRINITY DN31326 c5 g1TRINITY DN3132
TRINITY DN31326 c5 g1TRINITY DN31326 c5
TRINITY DN35401 c0 g1TRINITY DN3540
TRINITY DN35401 c0 g1TRINITY DN354
TRINITY DN35401 c0 g1TRINITY DN354
TRINITY DN6773 c0 g2TRINITY DN6773
TRINITY DN6773 c0 g1TRINITY DN6773 c
